# 3-Benzyl-6-methyl-2-sulfanylidene-2,3-di­hydroquinazolin-4(1*H*)-one

**DOI:** 10.1107/S1600536812006009

**Published:** 2012-02-17

**Authors:** Rashad Al-Salahi, Mohamed Al-Omar, Hussein El-Subbagh, Madhukar Hemamalini, Hoong-Kun Fun

**Affiliations:** a Department of Pharmaceutical Chemistry, College of Pharmacy, King Saud University, Riyadh 11451, Saudi Arabia; b Drug Exploration and Development Chair, College of Pharmacy, King Saud University, Riyadh 11451, Saudi Arabia; c College of Pharmaceutical Sciences, Future University, Cairo 12311, Egypt; d X-ray Crystallography Unit, School of Physics, Universiti Sains Malaysia, 11800 USM, Penang, Malaysia

## Abstract

In the title compound, C_16_H_14_N_2_OS, the quinazoline ring system is essentially planar, with a maximum deviation of 0.029 (3) Å. The dihedral angle between the quinazoline and benzene rings is 88.4 (2)°. In the crystal, adjacent mol­ecules are connected *via* pairs of N—H⋯S and C—H⋯O hydrogen bonds, which generate *R* ^2^_2_(8) and *R* ^2^_2_(10) graph-set motifs, respectively, resulting in a supra­molecular chain along the *a* axis.

## Related literature

For details and applications of quinazoline compounds, see: Roth & Fenner (2000 ▶); Jantova *et al.* (2004 ▶); Harris & Thorarensen (2004 ▶); Andries *et al.* (2005 ▶); Al-Rashood *et al.* (2006 ▶); Ghorab *et al.* (2007 ▶); Rádl *et al.* (2000 ▶); Klepser & Klepser (1997 ▶); Al-Omar *et al.* (2004 ▶); Al-Omary *et al.* (2010 ▶). For hydrogen-bond motifs, see: Bernstein *et al.* (1995 ▶). For bond-length data, see: Allen *et al.* (1987 ▶).
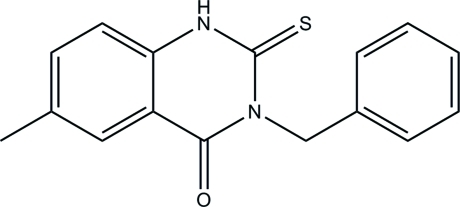


## Experimental

### 

#### Crystal data

C_16_H_14_N_2_OS
*M* *_r_* = 282.35
Monoclinic, 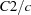

*a* = 24.2438 (18) Å
*b* = 5.1618 (5) Å
*c* = 24.4265 (17) Å
β = 111.532 (6)°
*V* = 2843.4 (4) Å^3^
*Z* = 8
Cu *K*α radiation
μ = 1.99 mm^−1^
*T* = 296 K
0.83 × 0.12 × 0.06 mm

#### Data collection

Bruker SMART APEXII CCD diffractometer
Absorption correction: multi-scan (*SADABS*; Bruker, 2009 ▶) *T*_min_ = 0.289, *T*_max_ = 0.890
9528 measured reflections
2592 independent reflections
1421 reflections with *I* > 2σ(*I*)
*R*_int_ = 0.104

#### Refinement

*R*[*F* ^2^ > 2σ(*F* ^2^)] = 0.055
*wR*(*F* ^2^) = 0.166
*S* = 0.93
2592 reflections
182 parameters
H-atom parameters constrained
Δρ_max_ = 0.24 e Å^−3^
Δρ_min_ = −0.21 e Å^−3^

### 

Data collection: *APEX2* (Bruker, 2009 ▶); cell refinement: *SAINT* (Bruker, 2009 ▶); data reduction: *SAINT*; program(s) used to solve structure: *SHELXTL* (Sheldrick, 2008 ▶); program(s) used to refine structure: *SHELXTL*; molecular graphics: *SHELXTL*; software used to prepare material for publication: *SHELXTL* and *PLATON* (Spek, 2009 ▶).

## Supplementary Material

Crystal structure: contains datablock(s) global, I. DOI: 10.1107/S1600536812006009/is5070sup1.cif

Structure factors: contains datablock(s) I. DOI: 10.1107/S1600536812006009/is5070Isup2.hkl

Supplementary material file. DOI: 10.1107/S1600536812006009/is5070Isup3.cml

Additional supplementary materials:  crystallographic information; 3D view; checkCIF report

## Figures and Tables

**Table 1:** Hydrogen-bond geometry (Å, °)

*D*—H⋯*A*	*D*—H	H⋯*A*	*D*⋯*A*	*D*—H⋯*A*
N1—H1*A*⋯S1^i^	0.86	2.50	3.335 (3)	165
C4—H4*A*⋯O1^ii^	0.93	2.41	3.295 (4)	159

Symmetry codes: (i) 
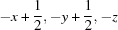
; (ii) 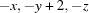.

**Fig. 1.**
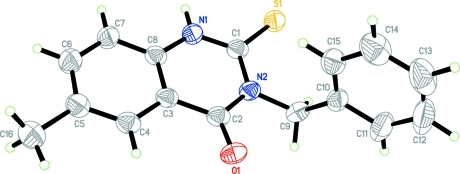
The molecular structure of the title compound, showing 30% probability displacement ellipsoids.

**Fig. 2.**
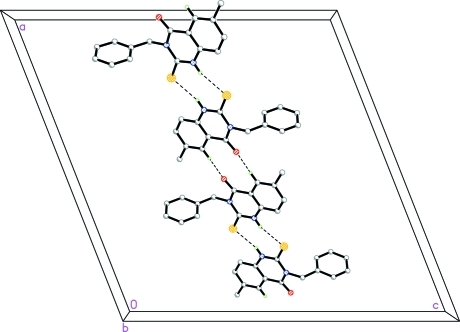
The crystal packing view of the title compound along the b axis.

## supplementary crystallographic information

### Comment

Quinazoline moiety is present in many classes of biologically-active compounds. A number of them have been clinically used as antifungal, antibacterial and antiprotozoic drugs (Roth & Fenner, 2000; Jantova *et al.*, 2004; Harris & Thorarensen, 2004), as well as antituberculotic agents (Andries *et al.*, 2005). Furthermore, they have drawn much attention due to their broad range of pharmacological properties which include antitumor (Al-Rashood *et al.*, 2006), anticancer (Ghorab *et al.*, 2007) and analgesic (Rádl *et al.*, 2000) properties. Certain quinazoline analogs also showed remarkable activity against the opportunistic infections of Pneumocystis carinii and Toxoplasma gondii. Those microorganisms proved to be the priniciple cause of death in patients with immunocompromised diseases such as acquired immune deficiency syndrome (Klepser & Klepser, 1997). This work is a continuation of this program with the aim of obtaining an interesting series of quinazolines that contain the thioxo functional group which was identified as a possible pharmacophore of the antimicrobial activity (Al-Omar *et al.*, 2004; Al-Omary *et al.*, 2010).

The molecular structure of the title compound is shown in Fig. 1. The quinazoline (N1,N2/C1–C8) ring is essentially planar, with a maximum deviation of 0.029 (3) Å for atom C2. The dihedral angle between the quinazoline (N1,N2/C1–C8) and the benzene (C10–C15) rings is 88.4 (2)°. The bond lengths (Allen *et al.*, 1987) and angles are within normal ranges. In the crystal, (Fig. 2), the adjacent molecules are connected *via* a pair of N—H···S and C—H···O (Table 1) hydrogen bonds, generating *R*^2^_2_(8) and *R*^2^_2_(10) graph-set motifs (Bernstein *et al.*, 1995), respectively, resulting in a supramolecular [100] chain.

### Experimental

A mixture of benzyl isothiocyanate (10 mmol) and 2-amino-5-methyl benzoic acid (10 mmol) in ethanol (30 ml) was heated under reflux in the presence of triethylamine (5 mmol) for 2 h. After cooling, the mixture was poured into ice/water. The resulting solid was filtered, washed with water and dried. Recrystallization from ethanol gave 3-benzyl-2,3-dihydro-6-methyl-2-thioxo-quinazoline-4(1*H*)-one as colorless crystals.

### Refinement

All H atoms were positioned geometrically (N—H = 0.86 Å and C—H = 0.93–0.97 Å) and were refined using a riding model, with *U*_iso_(H) = 1.2 or 1.5*U*_eq_(C). A rotating group model was applied to the methyl groups.

### Crystal data

**Table:**

C_16_H_14_N_2_OS	*F*(000) = 1184
*M**_r_* = 282.35	*D*_x_ = 1.319 Mg m^−^^3^
Monoclinic, *C*2/*c*	Cu *K*α radiation, λ = 1.54178 Å
Hall symbol: -C 2yc	Cell parameters from 339 reflections
*a* = 24.2438 (18) Å	θ = 3.9–53.5°
*b* = 5.1618 (5) Å	µ = 1.99 mm^−^^1^
*c* = 24.4265 (17) Å	*T* = 296 K
β = 111.532 (6)°	Needle, colourless
*V* = 2843.4 (4) Å^3^	0.83 × 0.12 × 0.06 mm
*Z* = 8	

### Data collection

**Table:**

Bruker SMART APEXII CCD diffractometer	2592 independent reflections
Radiation source: fine-focus sealed tube	1421 reflections with *I* > 2σ(*I*)
Graphite monochromator	*R*_int_ = 0.104
φ and ω scans	θ_max_ = 69.8°, θ_min_ = 3.9°
Absorption correction: multi-scan (*SADABS*; Bruker, 2009)	*h* = −29→29
*T*_min_ = 0.289, *T*_max_ = 0.890	*k* = −6→5
9528 measured reflections	*l* = −28→28

### Refinement

**Table:**

Refinement on *F*^2^	Primary atom site location: structure-invariant direct methods
Least-squares matrix: full	Secondary atom site location: difference Fourier map
*R*[*F*^2^ > 2σ(*F*^2^)] = 0.055	Hydrogen site location: inferred from neighbouring sites
*wR*(*F*^2^) = 0.166	H-atom parameters constrained
*S* = 0.93	*w* = 1/[σ^2^(*F*_o_^2^) + (0.0875*P*)^2^] where *P* = (*F*_o_^2^ + 2*F*_c_^2^)/3
2592 reflections	(Δ/σ)_max_ = 0.001
182 parameters	Δρ_max_ = 0.24 e Å^−^^3^
0 restraints	Δρ_min_ = −0.21 e Å^−^^3^

### Special details

**Table:**

Geometry. All s.u.'s (except the s.u. in the dihedral angle between two l.s. planes) are estimated using the full covariance matrix. The cell s.u.'s are taken into account individually in the estimation of s.u.'s in distances, angles and torsion angles; correlations between s.u.'s in cell parameters are only used when they are defined by crystal symmetry. An approximate (isotropic) treatment of cell s.u.'s is used for estimating s.u.'s involving l.s. planes.
Refinement. Refinement of F^2^ against ALL reflections. The weighted R-factor wR and goodness of fit S are based on F^2^, conventional R-factors R are based on F, with F set to zero for negative F^2^. The threshold expression of F^2^ > 2σ(F^2^) is used only for calculating R-factors(gt) etc. and is not relevant to the choice of reflections for refinement. R-factors based on F^2^ are statistically about twice as large as those based on F, and R-factors based on ALL data will be even larger.

### Fractional atomic coordinates and isotropic or equivalent isotropic displacement parameters (Å^2^)

**Table:**

	*x*	*y*	*z*	*U*_iso_*/*U*_eq_	
S1	0.22029 (4)	0.1447 (2)	0.07055 (4)	0.0718 (3)	
N1	0.17921 (10)	0.4985 (6)	−0.01167 (11)	0.0630 (7)	
H1A	0.2097	0.4590	−0.0199	0.076*	
N2	0.12318 (10)	0.4401 (5)	0.04598 (11)	0.0574 (7)	
O1	0.04534 (10)	0.7057 (5)	0.03541 (11)	0.0747 (7)	
C1	0.17206 (13)	0.3706 (7)	0.03308 (14)	0.0594 (8)	
C2	0.08423 (13)	0.6424 (7)	0.01808 (14)	0.0600 (8)	
C3	0.09395 (12)	0.7664 (7)	−0.03127 (13)	0.0585 (8)	
C4	0.05611 (13)	0.9575 (7)	−0.06444 (14)	0.0630 (9)	
H4A	0.0243	1.0100	−0.0544	0.076*	
C5	0.06438 (15)	1.0717 (7)	−0.11196 (15)	0.0691 (9)	
C6	0.11382 (16)	0.9891 (8)	−0.12434 (16)	0.0763 (10)	
H6A	0.1206	1.0638	−0.1559	0.092*	
C7	0.15210 (15)	0.8048 (7)	−0.09221 (15)	0.0715 (10)	
H7A	0.1848	0.7578	−0.1013	0.086*	
C8	0.14201 (13)	0.6879 (7)	−0.04573 (14)	0.0578 (8)	
C9	0.10920 (14)	0.2997 (7)	0.09172 (15)	0.0657 (9)	
H9A	0.1263	0.1276	0.0958	0.079*	
H9B	0.0665	0.2799	0.0788	0.079*	
C10	0.13111 (14)	0.4281 (7)	0.15093 (14)	0.0641 (9)	
C11	0.1103 (2)	0.3376 (10)	0.1930 (2)	0.0988 (15)	
H11A	0.0831	0.2022	0.1840	0.119*	
C12	0.1298 (3)	0.4483 (15)	0.2483 (2)	0.131 (2)	
H12A	0.1144	0.3902	0.2758	0.157*	
C13	0.1710 (3)	0.6401 (16)	0.2634 (2)	0.133 (2)	
H13A	0.1854	0.7057	0.3015	0.160*	
C14	0.1912 (2)	0.7364 (11)	0.2216 (2)	0.1082 (15)	
H14A	0.2179	0.8737	0.2306	0.130*	
C15	0.17129 (17)	0.6268 (8)	0.16567 (16)	0.0783 (11)	
H15A	0.1856	0.6898	0.1377	0.094*	
C16	0.02217 (18)	1.2711 (9)	−0.14931 (18)	0.0893 (12)	
H16B	−0.0064	1.3143	−0.1321	0.134*	
H16A	0.0021	1.2027	−0.1881	0.134*	
H16C	0.0438	1.4238	−0.1516	0.134*	

### Atomic displacement parameters (Å^2^)

**Table:**

	*U*^11^	*U*^22^	*U*^33^	*U*^12^	*U*^13^	*U*^23^
S1	0.0640 (5)	0.0875 (7)	0.0707 (6)	0.0172 (4)	0.0327 (5)	0.0084 (5)
N1	0.0558 (14)	0.083 (2)	0.0576 (17)	0.0153 (13)	0.0295 (14)	0.0029 (15)
N2	0.0510 (13)	0.0688 (18)	0.0577 (16)	0.0036 (11)	0.0264 (13)	−0.0049 (13)
O1	0.0601 (12)	0.0924 (19)	0.0855 (17)	0.0135 (11)	0.0430 (13)	−0.0007 (13)
C1	0.0500 (16)	0.076 (2)	0.0554 (19)	0.0024 (14)	0.0231 (16)	−0.0121 (17)
C2	0.0498 (16)	0.073 (2)	0.059 (2)	0.0022 (14)	0.0228 (16)	−0.0124 (17)
C3	0.0472 (16)	0.076 (2)	0.051 (2)	−0.0010 (14)	0.0169 (16)	−0.0104 (17)
C4	0.0543 (17)	0.070 (2)	0.066 (2)	0.0069 (15)	0.0225 (17)	−0.0068 (18)
C5	0.065 (2)	0.074 (2)	0.066 (2)	0.0057 (16)	0.0211 (18)	−0.0006 (19)
C6	0.077 (2)	0.086 (3)	0.072 (2)	0.0089 (18)	0.035 (2)	0.008 (2)
C7	0.068 (2)	0.090 (3)	0.068 (2)	0.0134 (17)	0.0375 (19)	0.009 (2)
C8	0.0472 (16)	0.073 (2)	0.0540 (19)	0.0062 (13)	0.0194 (15)	−0.0060 (16)
C9	0.0629 (19)	0.065 (2)	0.080 (2)	−0.0013 (14)	0.0393 (19)	−0.0001 (18)
C10	0.0643 (18)	0.074 (2)	0.063 (2)	0.0196 (16)	0.0343 (18)	0.0090 (18)
C11	0.115 (3)	0.114 (4)	0.090 (3)	0.020 (3)	0.065 (3)	0.028 (3)
C12	0.150 (6)	0.185 (7)	0.080 (4)	0.064 (5)	0.069 (4)	0.043 (4)
C13	0.131 (5)	0.196 (7)	0.068 (3)	0.061 (4)	0.031 (4)	−0.018 (4)
C14	0.108 (4)	0.117 (4)	0.092 (3)	0.010 (3)	0.027 (3)	−0.030 (3)
C15	0.082 (2)	0.089 (3)	0.066 (3)	0.002 (2)	0.029 (2)	−0.011 (2)
C16	0.082 (3)	0.091 (3)	0.092 (3)	0.014 (2)	0.028 (2)	0.012 (2)

### Geometric parameters (Å, º)

**Table:**

S1—C1	1.667 (3)	C7—H7A	0.9300
N1—C1	1.342 (4)	C9—C10	1.500 (5)
N1—C8	1.382 (4)	C9—H9A	0.9700
N1—H1A	0.8600	C9—H9B	0.9700
N2—C1	1.381 (3)	C10—C15	1.369 (5)
N2—C2	1.405 (4)	C10—C11	1.381 (5)
N2—C9	1.472 (4)	C11—C12	1.382 (7)
O1—C2	1.212 (3)	C11—H11A	0.9300
C2—C3	1.458 (4)	C12—C13	1.357 (9)
C3—C4	1.388 (5)	C12—H12A	0.9300
C3—C8	1.396 (4)	C13—C14	1.377 (8)
C4—C5	1.381 (5)	C13—H13A	0.9300
C4—H4A	0.9300	C14—C15	1.390 (6)
C5—C6	1.406 (5)	C14—H14A	0.9300
C5—C16	1.501 (5)	C15—H15A	0.9300
C6—C7	1.359 (5)	C16—H16B	0.9600
C6—H6A	0.9300	C16—H16A	0.9600
C7—C8	1.385 (4)	C16—H16C	0.9600
			
C1—N1—C8	126.0 (2)	N2—C9—C10	114.6 (3)
C1—N1—H1A	117.0	N2—C9—H9A	108.6
C8—N1—H1A	117.0	C10—C9—H9A	108.6
C1—N2—C2	124.2 (3)	N2—C9—H9B	108.6
C1—N2—C9	120.1 (3)	C10—C9—H9B	108.6
C2—N2—C9	115.7 (2)	H9A—C9—H9B	107.6
N1—C1—N2	115.9 (3)	C15—C10—C11	118.5 (4)
N1—C1—S1	121.1 (2)	C15—C10—C9	123.4 (3)
N2—C1—S1	123.0 (2)	C11—C10—C9	118.1 (4)
O1—C2—N2	120.1 (3)	C10—C11—C12	120.0 (5)
O1—C2—C3	123.6 (3)	C10—C11—H11A	120.0
N2—C2—C3	116.3 (2)	C12—C11—H11A	120.0
C4—C3—C8	119.5 (3)	C13—C12—C11	121.3 (5)
C4—C3—C2	121.5 (3)	C13—C12—H12A	119.3
C8—C3—C2	119.0 (3)	C11—C12—H12A	119.3
C5—C4—C3	121.7 (3)	C12—C13—C14	119.3 (5)
C5—C4—H4A	119.1	C12—C13—H13A	120.4
C3—C4—H4A	119.1	C14—C13—H13A	120.4
C4—C5—C6	116.7 (3)	C13—C14—C15	119.5 (6)
C4—C5—C16	121.8 (3)	C13—C14—H14A	120.3
C6—C5—C16	121.5 (3)	C15—C14—H14A	120.3
C7—C6—C5	122.9 (3)	C10—C15—C14	121.3 (4)
C7—C6—H6A	118.5	C10—C15—H15A	119.3
C5—C6—H6A	118.5	C14—C15—H15A	119.3
C6—C7—C8	119.3 (3)	C5—C16—H16B	109.5
C6—C7—H7A	120.3	C5—C16—H16A	109.5
C8—C7—H7A	120.3	H16B—C16—H16A	109.5
N1—C8—C7	122.0 (3)	C5—C16—H16C	109.5
N1—C8—C3	118.3 (3)	H16B—C16—H16C	109.5
C7—C8—C3	119.7 (3)	H16A—C16—H16C	109.5
			
C8—N1—C1—N2	−0.9 (5)	C1—N1—C8—C7	−178.2 (3)
C8—N1—C1—S1	179.7 (3)	C1—N1—C8—C3	3.6 (5)
C2—N2—C1—N1	−4.2 (5)	C6—C7—C8—N1	179.7 (4)
C9—N2—C1—N1	176.0 (3)	C6—C7—C8—C3	−2.1 (6)
C2—N2—C1—S1	175.1 (2)	C4—C3—C8—N1	179.5 (3)
C9—N2—C1—S1	−4.6 (4)	C2—C3—C8—N1	−1.3 (5)
C1—N2—C2—O1	−173.6 (3)	C4—C3—C8—C7	1.3 (5)
C9—N2—C2—O1	6.1 (5)	C2—C3—C8—C7	−179.6 (3)
C1—N2—C2—C3	6.2 (5)	C1—N2—C9—C10	97.0 (3)
C9—N2—C2—C3	−174.1 (3)	C2—N2—C9—C10	−82.7 (3)
O1—C2—C3—C4	−4.2 (5)	N2—C9—C10—C15	−13.4 (5)
N2—C2—C3—C4	176.0 (3)	N2—C9—C10—C11	167.7 (3)
O1—C2—C3—C8	176.6 (3)	C15—C10—C11—C12	0.3 (6)
N2—C2—C3—C8	−3.1 (5)	C9—C10—C11—C12	179.3 (4)
C8—C3—C4—C5	0.5 (5)	C10—C11—C12—C13	−2.3 (8)
C2—C3—C4—C5	−178.6 (3)	C11—C12—C13—C14	3.8 (10)
C3—C4—C5—C6	−1.4 (5)	C12—C13—C14—C15	−3.2 (8)
C3—C4—C5—C16	177.6 (3)	C11—C10—C15—C14	0.2 (6)
C4—C5—C6—C7	0.5 (6)	C9—C10—C15—C14	−178.7 (4)
C16—C5—C6—C7	−178.5 (4)	C13—C14—C15—C10	1.3 (7)
C5—C6—C7—C8	1.3 (6)		

### Hydrogen-bond geometry (Å, º)

**Table:**

*D*—H···*A*	*D*—H	H···*A*	*D*···*A*	*D*—H···*A*
N1—H1*A*···S1^i^	0.86	2.50	3.335 (3)	165
C4—H4*A*···O1^ii^	0.93	2.41	3.295 (4)	159

Symmetry codes: (i) −*x*+1/2, −*y*+1/2, −*z*; (ii) −*x*, −*y*+2, −*z*.
